# Antioxidant Potential of Mung Bean (*Vigna radiata*) Albumin Peptides Produced by Enzymatic Hydrolysis Analyzed by Biochemical and In Silico Methods

**DOI:** 10.3390/foods9091241

**Published:** 2020-09-04

**Authors:** Jennifer Kusumah, Luis M. Real Hernandez, Elvira Gonzalez de Mejia

**Affiliations:** Department of Food Science and Human Nutrition, University of Illinois at Urbana-Champaign, Urbana, IL 61801, USA; kusumah2@illinois.edu (J.K.); rhealmlus@gmail.com (L.M.R.H.)

**Keywords:** albumin, albumin peptide, antioxidant peptide, bioactive peptide, in silico, mung bean, mung bean albumin, peptide sequencing, *Vigna radiata*

## Abstract

The objective of this study was to investigate the biochemical antioxidant potential of peptides derived from enzymatically hydrolyzed mung bean (*Vigna radiata)* albumins using an 2,2′-Azino-bis(3-ethylbenzothiazoline-6-sulfonic acid) (ABTS) radical scavenging assay, a ferrous ion chelating assay and an oxygen radical absorbance capacity (ORAC) assay. Peeled raw mung bean was ground into flour and mixed with buffer (pH 8.3, 1:20 *w/v* ratio) before being stirred, then filtered using 3 kDa and 30 kDa molecular weight cut-off (MWCO) centrifugal filters to obtain albumin fraction. The albumin fraction then underwent enzymatic hydrolysis using either gastrointestinal enzymes (pepsin and pancreatin) or thermolysin. Peptides in the hydrolysates were sequenced. The peptides showed low ABTS radical-scavenging activity (90–100 μg ascorbic acid equivalent/mL) but high ferrous ion chelating activity (1400–1500 μg EDTA equivalent/mL) and ORAC values (>120 μM Trolox equivalent). The ferrous ion chelating activity was enzyme- and hydrolysis time-dependent. For thermolysin hydrolysis, there was a drastic increase in ferrous ion chelating activity from t = 0 (886.9 μg EDTA equivalent/mL) to t = 5 min (1559.1 μg EDTA equivalent/mL) before plateauing. For pepsin–pancreatin hydrolysis, there was a drastic decrease from t = 0 (878.3 μg EDTA equivalent/mL) to t = 15 (138.0 μg EDTA equivalent/mL) after pepsin was added, but this increased from t = 0 (131.1 μg EDTA equivalent/mL) to t = 15 (1439.2 μg EDTA equivalent/mL) after pancreatin was added. There was no significant change in ABTS radical scavenging activity or ORAC values throughout different hydrolysis times for either the thermolysin or pepsin–pancreatin hydrolysis. Overall, mung bean hydrolysates produced peptides with high potential antioxidant capacity, being particularly effective ferrous ion chelators. Other antioxidant assays that use cellular lines should be performed to measure antioxidant capacity before animal and human studies.

## 1. Introduction

Mung bean, also known as green gram, is a small, green-colored legume widely cultivated throughout Asia [1]. It is a popular legume in countries such as Indonesia and China where its consumption is associated with positive health outcomes [2,3]. Mung bean flour is commonly made into a paste and incorporated into bread and desserts [4]. Mung beans have a relatively high protein content (19.5–33.1%) that is comparable to that of soybeans (*Glycine max*) (35–50%) and kidney beans (*Phaseolus vulgaris*) (23–25%) [5,6,7]. Due to its nutritional content, mung bean can be a plant-based protein source in developing nations where animal protein sources are cost-prohibited [8]. Compared to other legumes, mung beans are relatively free from antinutritional factors [9]. Mung beans are also rich in vitamins and minerals such as iron, magnesium, potassium, copper and folate [10].

**Figure 1:**
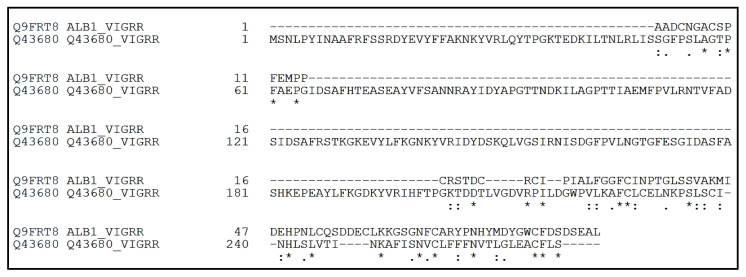
Alignment of the mung bean albumin sequences currently present in the UniProt Database. The alignment was conducted using Clustal Omega. Q9FRT8 is the reviewed sequence of a 10 kDa protein fragment, while Q43680 is the non-reviewed sequence of a 30 kDa protein. The sequences have 7% identity, with 19 identical positions and 21 similar positions. Asterisks (*) indicate identical amino acids in the sequences, while dots indicate amino acids in the sequences that are not identical.

The major storage proteins of mung bean are globulins (62.0%), albumins (16.3%), glutelins (13.3%) and prolamins (0.9%), with vicilin-type protein (8S) making up 89% of globulins [11]. Globulins are the main storage proteins in mature mung beans, and they are also the most well studied mung bean proteins [12]. In contrast to globulins, there are limited studies on the albumin proteins of mung beans [13]. Albumins are water-soluble, globular proteins found in both animals and plants [14]. In plants, albumins can be proteins stored in seeds to be used during germination and growth [15]. The isolation and characterization of some albumins in multiple legumes, such as lentils, soybeans and winged beans, has already been performed [16,17,18]. There are currently two main entries for mung bean albumin in the UniProt Database; Q9FRT8 is a reviewed entry detailing a 10 kDa protein fragment. Q43680 is a non-reviewed entry detailing a 30 kDa protein. The sequences of these two entries are given in Figure 1, and the sequences have minimal similarity (7% identity). There is currently no evidence that only a single mung bean albumin exists, so reported mung bean albumin sequences can be different from each other. Given the current data, mung bean albumins are not expected to have a molecular weight greater than 30 kDa [19,20].

Peptides derived from chickpea albumins, which are legume albumins like those present in mung beans, have shown high biologically relevant antioxidant potential [21,22]. Antioxidant peptides can benefit human health by chelating excess transition metal ions and scavenging free radicals and reactive oxygen species [23]. Lunasin, a peptide derived from soybean 2S albumin, has also been found to have antioxidant effects [24,25]. Peptides from whole mung bean protein hydrolysates have been found to have calcium and ferrous ion binding activity that can have biological implications, but this activity is also useful in preventing oxidation in food systems [26]. However, the antioxidant potential of mung bean albumin hydrolysates and peptides alone, without the presence of other mung bean proteins, has been poorly studied [27]. The objective for this study was to investigate the biochemical antioxidant potential of peptides derived from enzymatically hydrolyzed mung bean albumins using either thermolysin or gastrointestinal enzymes pepsin and pancreatin, followed by sequencing and characterizing the peptides. Antioxidant potential was investigated using an ABTS radical scavenging assay, a ferrous ion chelating assay and ORAC assay.

## 2. Materials and Methods

### 2.1. Materials

Peeled and split raw mung bean dry seeds were purchased locally (Asian Best, Thailand), and stored at 4 °C. The purchased mung beans were from a single brand cultivated in Thailand. The beans were taken from 4 °C bulk storage for each experimental sampling, at least three times for each experiment performed.

Centrifugal ultrafiltration filters with 3 or 30 kDa MWCO membranes were purchased from Millipore-Sigma (St. Louis, MO, USA). Protein reagents A and B, 2× Laemmli sample buffer, 10× tris/glycine and 10× tris/glycine/SDS buffers, mini-PROTEAN^®^ TGX™ gels, Coomassie blue and Precision Plus Protein™ Dual Xtra standard were purchased from Bio-Rad (Hercules, CA, USA).

Simply Blue Safe Stain was purchased from Invitrogen (Carlsbad, CA, USA). All other reagents were purchased from Sigma-Aldrich (St. Louis, MO, USA) unless stated otherwise.

### 2.2. Mung Bean Albumin Extraction

Peeled dry mung beans were milled and sieved using a number 35 mesh (500 μm). Collected flour was stored at 4 °C until use. Since a standard extraction protocol for mung bean albumins has not been established, two different legume albumin extraction methods were implemented to extract mung bean albumins.

For one method, a recently proposed protocol for the extraction of mung bean albumins was followed as specified in Du et al. [28] with specific modifications. The collected mung bean flour was mixed with double-distilled water at a ratio of 1:20 (*w/v*). The pH of the mixture was adjusted to either 3.0 or 7.5 with 1 M HCl or NaOH, respectively. The extraction pH values used were previously found to solubilize a higher concentration of mung bean albumins compared to total mung bean proteins [28]. Albumins were extracted for 1 h at 25 °C with manual stirring of each mixture every 15 min. The mixtures were then centrifuged at 11,000× *g* for 20 min at 4 °C, and the collected supernatants had their pH adjusted to 4.6 using 0.1 M HCl or NaOH. The supernatants at pH 4.6 were centrifuged at 11,000× *g* for 20 min at 4 °C, and the precipitated mung bean albumin pellets were collected. The mung bean albumin pellets for each extraction pH were solubilized individually in 5 mL of double-distilled water (pH = 11) and sonicated for 1 min to facilitate solvation. The resulting solutions were filtered using 30 kDa MWCO ultrafiltration centrifugal filters by centrifuging the solutions at 6600× *g* for 1 h at 4 °C. The resulting permeate was collected and used as an aqueous filtrate of extracted mung bean albumins. The collected permeates were stored at 4 °C for ≤3 days.

The other extraction method followed the protocol published by Singh, Rao and Singh [29] with minor adaptations. Collected mung bean flour was mixed with 0.1 M borate buffer (pH = 8.3) at a ratio of 1:20 (*w/v*) and was continuously stirred for 1 h. The mixture was then centrifuged at 11,000× *g* for 20 min at 4 °C, and the supernatant was collected. As an alternative to extensive dialysis, the collected supernatant was filtered using a 3 kDa MWCO ultrafiltration centrifugal filter at 6600× *g* for 1 h at 4 °C, and then the retentate was supplied with sodium citrate buffer (pH = 4.6) to replenish the buffer volume lost as permeate. The solution at pH 4.6 was centrifuged at 11,000× *g* for 20 min at 4 °C, and the collected supernatant was filtered using a 30 kDa MWCO ultrafiltration centrifugal filter at 6600× *g* for 1 h at 4 °C. The resulting permeate was collected and used as an aqueous filtrate of extracted mung bean albumins. The collected permeates were stored at 4 °C for ≤3 days

### 2.3. Gel Electrophoresis

#### 2.3.1. Sodium Dodecyl Sulfate–Polyacrylamide Gel Electrophoresis (SDS-PAGE)

The DC protein assay was used to determine the protein concentrations of mung bean albumin samples using a bovine serum albumin standard curve. SDS-PAGE was performed as published before with minor modifications [30]. Briefly, mung bean samples were mixed with 2× Laemmli buffer (1:1 *v/v*) without 2-mercaptoethanol and boiled for 5 min. The prepared samples were loaded on 4–20% Tris-glycine gels so that each well had 20 μg of crude mung bean albumins (unfiltered extract) or 15 μg of filtered mung bean albumins (≤30 kDa permeate). A Tris-glycine buffer consisting of 25 mM Tris and 192 mM glycine at pH 8.3 was used in both the anode and cathode, and the gels ran at 200 V for 30 min. SimplyBlue Safe Stain was used to stain the gels.

#### 2.3.2. Native Blue–Polyacrylamide Gel Electrophoresis (Native Blue-PAGE)

The SDS-PAGE methodology mentioned above was modified to run native blue gels. Mung bean samples were mixed (1:1 *v/v*) with Tris-glycine buffer containing 25% glycerol. Prepared samples were loaded on 4–20% Tris-glycine gels so that each well had 20 μg of crude mung bean albumins (unfiltered extract) or 15 μg of filtered mung bean albumins (≤30 kDa permeate). Tris-glycine buffer was used in the anode, and Tris-glycine buffer containing 2% Coomassie blue dye was used in the cathode. The gels ran at 150 V for 1 h at 22 °C. The gels were de-stained with a 25% isopropanol solution (*v/v*) containing 10% acetic acid (*v/v*).

### 2.4. In Silico Hydrolysis of Mung Bean Albumin Sequences

To determine proteases, outside of those present during gastrointestinal digestion, capable of potentially producing antioxidant peptides from mung bean albumins, a manual theoretical hydrolysis of the two reported mung bean albumin sequences (Q9FRT8 and Q43680) present in the UniProt Database (https://www.uniprot.org/) was performed. The theoretical hydrolysis was carried out using the protease specificity data present in the MEROPS Database (https://www.ebi.ac.uk/merops/). Alcalase, stem bromelain, ficin, papain and thermolysin were selected for the theoretical hydrolysis as they are food-safe enzymes capable of being used in the commercial hydrolysis of mung bean albumins.

For each mung bean albumin sequence, fragments of ≤8 adjacent amino acids, corresponding to an amino acid sequence that could occupy sites P4-P4′ in the active site of a protease, were matched to the possible amino acids known to be present at the P4-P4′ sites of a protease when a protein substrate is hydrolyzed by that protease. The specificity of each protease analyzed is detailed in Table 1. If a fragment sequence matched the possible amino acids that would lead the sequence to be hydrolyzed by a specific protease, the corresponding P1 and P1′ amino acids in the fragment sequence were color-coded, as this is where the sequence would be expected to be hydrolyzed. Amino acids in the mung bean albumin sequences were color-coded either red or purple where hydrolysis would be expected to happen, and hydrolysis was expected to be possible between adjacent amino acids in the sequence that were color-coded the same color. Possible mung bean albumin peptide sequences that would be expected to be produced by each protease were obtained by cutting each mung bean sequence between two adjacent amino acids that were color-coded the same to produce fragments of various amino acid quantities. For conciseness and applicability, only possible di- and tri-peptides that could be produced for each protease were accounted for, as these small peptides have intestinal transporters that make them more bioavailable than their larger counterparts [31]. The bioactive fragments present in the two mung bean albumin sequences hydrolyzed were identified using the Database of Bioactive Peptides—BIOPEP Database (http://www.uwm.edu.pl/biochemia/index.php/pl/biopep).

### 2.5. Enzymatic Hydrolysis

The simulated gastrointestinal digestion of mung bean albumins was carried out using conditions used previously by Mojica and de Mejia [32]. Briefly, pepsin was added to a solution (pH = 2) of mung bean albumins (≤30 kDa fraction) at a ratio of 1:20 *w*/*w* for 2 h at 37 °C. After 2 h, pancreatin was added to the solution at a pepsin–mung bean albumin ratio of 1:20 *w*/*w*. The pH of the solution was increased to 7.5 using 1 M NaOH, and the solution was incubated for another 2 h at 37 °C. Enzymatic reactions were stopped by heating the solution to 90 °C for 15 min. The inactivated hydrolysate was stored at 4 °C for ≤2 days.

Thermolysin hydrolysis was carried out by adding thermolysin to a mung bean albumin solution (≤30 kDa fraction) at a thermolysin–mung bean albumin ratio of 1:20 *w*/*w*. The pH of the solution was adjusted to 8.0 using 1 M NaOH, and then the mixture was incubated at 70 °C for 4 h. Afterwards, the solution was heated to 95 °C for 15 min to stop proteolytic activity. The inactivated hydrolysate was stored at 4 °C for ≤2 days.

### 2.6. Antioxidant Assays

All antioxidant assays were carried out on fresh hydrolysates produced within ≤2 days of storage before the assays were conducted. Radical scavenging and ferrous ion chelating activities were calculated based on equations obtained from the standard curves using ascorbic acid and EDTA solutions, respectively.

#### 2.6.1. ABTS Radical Scavenging Assay

The ABTS radical scavenging assay was performed as published before for a mung bean meal hydrolysate [26]. A 7 mM ABTS and 2.5 mM potassium persulphate stock solution in 10 mM PBS buffer (pH = 7.4) was made and stored in darkness for 16 h. The stock solution was then diluted with 10 mM PBS buffer until an absorbance of 0.70 ± 0.05 at 734 nm was obtained. A sample of mung bean albumins (20 μL, 1 g/L) was added to 1980 μL of diluted ABTS solution. The mung bean albumin concentration was determined by the DC protein assay using a bovine serum albumin (BSA) standard curve, and preliminary studies were performed to determine the concentration used. The reaction was allowed to react for 5 min in the dark, and the resulting absorbance was read at 734 nm. The results were expressed as ascorbic acid equivalent in μM.

#### 2.6.2. Ferrous Ion Chelation Assay

The chelating activity of ferrous ions by mung bean peptides was analyzed as published before for a mung bean meal hydrolysate [26]. Mung bean albumins (100 μL, 1 g/L) were mixed with 100 μL of a 2 mM iron chloride solution, and the mixture was diluted with 1400 μL of double distilled water. Mung bean albumin concentration was determined by the DC protein assay using a bovine serum albumin (BSA) standard curve, and preliminary studies were performed to determine the concentration used. The mixture was incubated for 3 min at 25 °C. Afterwards, 400 μL of a 5 mM ferrozine solution was added, and the solution was incubated for 10 min at 25 °C. EDTA was used as a standard. The resulting absorbance was read at 562 nm. The result was expressed as EDTA equivalent in μM.

#### 2.6.3. Oxygen Radical Absorbance Capacity (ORAC) Assay

An ORAC assay on mung bean albumin hydrolysates was performed with some modification as published before [33]. Briefly, 20 μL of the sample (1 g/L) was mixed with 120 μL of fluorescein (0.12 mM). The absorbance was then read at 485 nm and the mixture was incubated at 37 °C for 15 min. A total of 60 μL of 40 mM 2′2-Azobis(2-amidinopropane) dihydrochloride (AAPH) was then added, and the solution was read again at 582 nm. Trolox was used as standard. The results were calculated using Equations (1) and (2) and reported as Trolox equivalent (μM):

(1) $$AUC=\left(\frac{R_{1}}{R_{1}}\right)+\;\left(\frac{R_{2}}{R_{1}}\right)\;+\;\left(\frac{R_{3}}{R_{1}}\right)+\ldots +\;\left(\frac{R_{n}}{R_{1}}\right)\;$$

(2) $$Net\;AUC=\;AUC_{sample}-\;AUC_{blank}$$

### 2.7. Peptide Sequencing

Peptide sequencing was performed as published before [34]. Briefly, peptides obtained from the different hydrolysates were analyzed by HPLC–ESI–MS/MS using a Q-ToF Ultima mass spectrometer (Waters, Milford, MA, USA) equipped with an Alliance 2795 HPLC system. The gradient mobile phase A was 95% water, 5% acetonitrile and 0.01% formic acid, while mobile phase B was 95% acetonitrile, 5% water and 0.1% formic acid. The volume of injection was 400 μL/min and the PDA detector wavelength was 280 nm. Each peak was analyzed in MassLynx V4.1 software (Waters Corp., Milford, MA, USA) and the sequence of amino acids was identified based on the accurate mass measurements, while tandem MS fragmentation using the MassBank database was used to analyze the data and obtain the peptide sequences with >80% of certainty. The isoelectric point, net charge and hydrophobicity of the peptides were analyzed by PepDraw [35]. The amino acids were presented as one letter nomenclature.

### 2.8. Statistical Analysis

The experiments were repeated at least three independent times from different beans, starting from the mung bean albumin extraction and proceeding up to the antioxidant assays and peptide sequencing. Data are expressed as mean ± standard deviation. The data obtained were analyzed using one-way ANOVA to compare experimental to control values, and differences were considered significant at *p* < 0.05. GraphPad Prism 7 (GraphPad Software, LLC., San Diego, CA, USA) was used for the data analysis.

## 3. Results

### 3.1. Mung Bean Albumin Profiles

**Figure 2:**
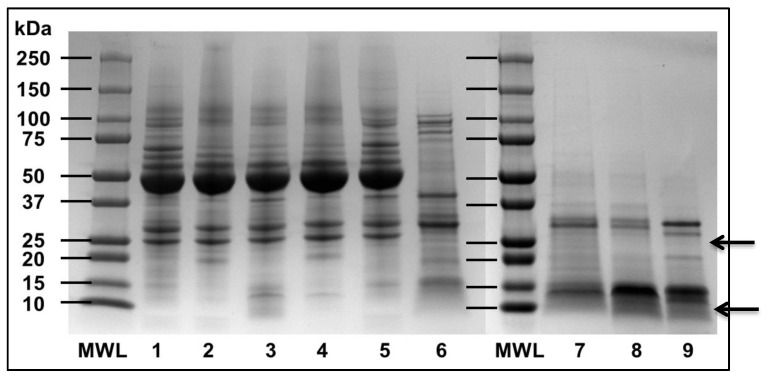
SDS-PAGE (Sodium Dodecyl Sulfate–Polyacrylamide Gel Electrophoresis) of mung bean albumin extracts and 30 kDa MWCO (molecular weight cut-off) filtrates. MWL = molecular weight ladder. (**1**) Water pH = 7.5 extract, (**2**) Albumin isolate from water pH = 7.5 extract, (**3**) Water pH = 3.0 extract, (**4**) Albumin isolate from water pH = 3.0 extract, (**5**) Borate buffer pH = 8.3 extract, (**6**) Albumin isolate from borate buffer pH = 8.3 extract. Samples on lanes (**2**), (**4**) and (**6**) were filtered using a 30 kDa MWCO centrifugal filter to produce the permeates on (**7**), (**8**) and (**9**), respectively. Arrows indicate the protein fractions that are most likely to be mung bean albumin according to their molecular weight.

**Figure 3:**
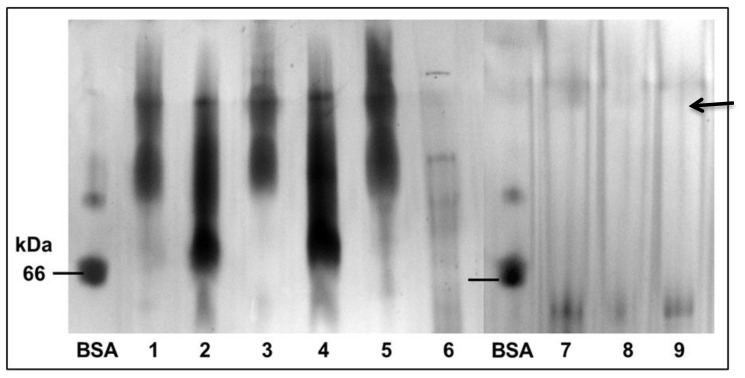
The native Blue-PAGE of mung bean albumin extracts and 30 kDa MWCO filtrates. BSA = bovine serum albumin. BSA was used as a reference protein. (**1**) Water pH = 7.5 extract, (**2**) Albumin isolate from water pH = 7.5 extract, (**3**) Water pH = 3.0 extract, (**4**) Albumin isolate from water pH = 3.0 extract, (**5**) Borate buffer pH = 8.3 extract, (**6**) Albumin isolate from borate buffer pH = 8.3 extract. Samples on lanes (**2**), (**4**) and (**6**) were filtered using a 30 kDa MWCO centrifugal filter to produce the permeates on (**7**), (**8**) and (**9**), respectively. Arrows indicate the protein fractions that are most likely to be mung bean albumin according to their molecular weight.

The mung bean albumin extraction methodologies used in this study resulted in mung bean albumin extracts with different protein profiles (Figure 2 and Figure 3). Only the albumin isolate obtained from mung bean albumins with borate buffer pH 8.3 had no 40–50 kDa globulins (Figure 2, lane 6). Therefore, the albumin isolate obtained from the borate buffer pH 8.3 extraction methodology was determined to be purer than the other protein isolates analyzed. Since mung bean albumins are not currently known to be larger than 30 kDa, the 30 kDa permeate of the albumin isolate obtained from the borate buffer pH 8.3 extraction protocol was sequenced. In addition, it was used for all antioxidant assays performed, as this isolate was determined to be the most consistent with the current literature [19,20]. The protein profile of this permeate is given on lane 9 of Figure 2.

### 3.2. Potentially Antioxidant Peptides from Mung Bean Albumins by In Silico Hydrolysis

**Figure 4:**
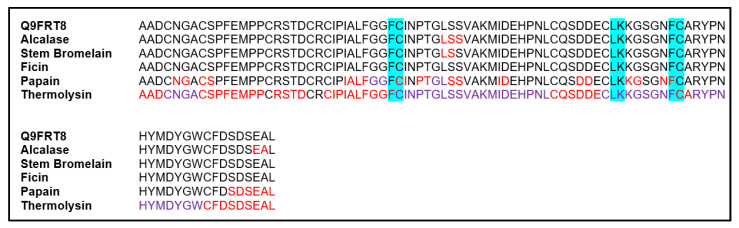
Locations of possible hydrolysis, by proteases selected, within the mung bean albumin sequence Q9FRT8 of the UniProt Database. Amino acids in the sequence are coded using their one letter abbreviation. Hydrolysis is expected to occur between any two adjacent amino acids color-coded the same color (red or purple), but not between amino acids color-coded with different colors or in black text. Each block represents a part of the sequence depicting the different specificities of the proteases stated in the left column. The top block is the start of the sequence. Antioxidant fragments according to the BIOPEP Database in the sequence are highlighted in blue.

**Figure 5:**
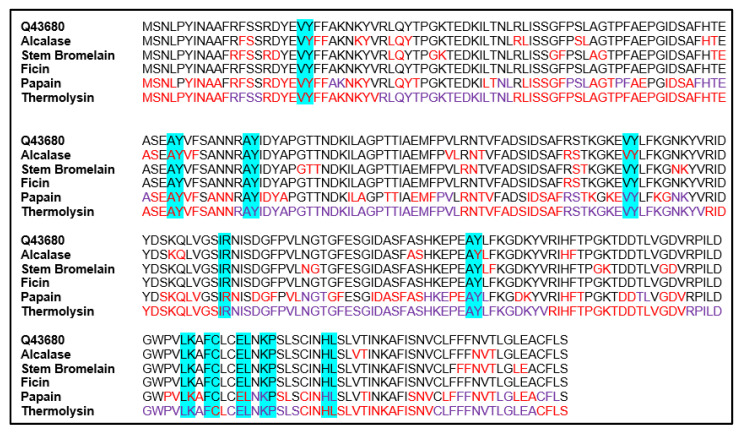
Locations of possible hydrolysis, by proteases selected, within the mung bean albumin sequence Q43680 of the UniProt Database. Amino acids in the sequence are coded using their one letter abbreviation. Hydrolysis is expected to occur between any two adjacent amino acids color-coded the same color (red or purple), but not between amino acids color-coded with different colors or in black text. Each block represents a part of the sequence depicting the different specificities of the proteases stated in the left column. The top block is the start of the sequence. Antioxidant fragments according to the BIOPEP Database in the sequence are highlighted in blue.

For the 10 kDa mung bean albumin sequence Q9FRT8, the bioactive fragments LK and FC were found for the sequence using the BIOPEP Database. These fragments were found in the Q9FRT8 sequence three times, and their locations are shown in Figure 4. For the 30 kDa mung bean albumin sequence Q43680, the bioactive fragments HL, AY, EL, IR, LK, KP, VY and FC were found using the BIOPEP Database. These fragments were found in the Q43680 sequence 11 times, and their locations are shown in Figure 5.

Thermolysin was found to be the protease most likely to produce small bioactive di-/tri-peptides from both mung bean albumin sequences in the UniProt Database analyzed (Table 2). Thermolysin was also found to be the enzyme most likely to destroy bioactive fragments in the mung bean albumin sequences analyzed, but given that it could hydrolyze the majority of the mung bean albumin sequences, this was expected (Figure 4 and Figure 5). Thermolysin was selected to hydrolyze mung bean albumins in vitro due to its greater potential to produce antioxidant peptides.

### 3.3. Antioxidant Activity of Mung Bean Albumin Hydrolysates and Peptide Sequencing

Results in Table 3 and Figure S1 show that mung bean albumins had some antioxidant capacity themselves (t = 0). Thermolysin and pepsin–pancreatin hydrolysis increased the antioxidant capacity in terms of ferrous ion chelating activity with significant changes due to time of hydrolysis. The ferrous ion chelating activity of the hydrolysates derived via both thermolysin and pepsin–pancreatin enzymatic hydrolysis was generally higher than the ABTS radical scavenging activity. ABTS and ORAC values did not present a significant increase in the antioxidant activities of any of the hydrolysates produced by the enzymes tested. The ferrous ion chelating activities of thermolysin and gastrointestinal peptides hydrolysates were not statistically different at the end of the hydrolysis. The same pattern was observed for ORAC values between thermolysin and gastrointestinal peptides. There was, however, significant difference between t = 0 and the rest of the time points for the ferrous ion chelating activity of thermolysin-derived peptides, except for pepsin, where the activity decreased. There was no significant difference in the ABTS radical scavenging activity at different time points for both thermolysin-derived and simulated gastrointestinal digestion-derived peptides.

Results in Table 4 and Table 5 also show that the peptides derived from enzymatic hydrolysis using gastrointestinal enzymes (pepsin-pancreatin), and with antioxidant potential, have higher molecular mass (~328 Da) compared to peptides derived from enzymatic hydrolysis using thermolysin (~253 Da). Enzymatic hydrolysis using thermolysin also produced more peptides with high hydrophobicity (>10 kcal/mol). All the peptides reported in Table 4 and Table 5 have been found to have antioxidant potential according to the BIOPEP Database, which is compounded from previously published literature.

Supplementary Materials Table S1 presents the complete sequences and functional properties of mung bean albumin hydrolysates derived from gastrointestinal enzymatic hydrolysis, and the effect of thermolysin. The values of the peptides found ranged between 236 and 1509 kDa, 5.81 and 20.74 Kcal/mol and 2.82 and 11.18 for molecular mass, hydrophobicity and isoelectric point, respectively. Supplementary Materials Table S2 presents the peptide sequences, functional properties and bioactivities of mung bean albumin hydrolysates derived from thermolysin enzymatic hydrolysis.

## 4. Discussion

A defining characteristic of mung bean albumins is their high solubility in water, but extracting them with water alone was found to be inadequate to isolate them from other mung bean proteins, as demonstrated by the SDS-PAGE result in Figure 2. The extraction of mung bean albumins using sodium borate buffer (pH = 8.3) and then subsequent centrifugal filtration was more effective for obtaining isolated mung bean albumins that were within the currently known filtration 3–30 kDa mass range. At pH 7.0, other mung bean proteins aside from albumins were soluble [28], and therefore the extraction of mung bean albumin with water alone at pH 7.0 will inevitably induce the extraction of other proteins. The solubility of other mung bean proteins lowered as the pH became higher than 7.0, while the solubility of albumin increased as the pH neared 8.0. Thus, using a buffer of pH 8.3 to extract mung bean albumins was more effective than using pure water, as the difference in solubility between albumin and the rest of the proteins at that pH allowed mung bean albumins to be effectively isolated. Mung bean albumin peptides produced through hydrolysis by thermolysin and gastrointestinal enzymes showed antioxidant potential. Mung bean albumin peptides showed high ferrous ion chelating activity, but low ABTS radical scavenging activity (Supplementary Materials Figure S1). The ferrous ion chelating activity of mung bean albumin peptides derived from pepsin–pancreatin enzymatic hydrolysis was found to be higher than the ferrous ion chelating activity of cowpea and common bean protein hydrolysates derived from the same enzyme scheme, as reported by Segura-Campos et al. [36]. The ferrous ion chelating activity of mung bean albumin peptides was also found to be higher than the ferrous ion chelating activity of soybean lunasin, as reported by Garcia-Nebot et al. [37]. It was also found to have higher ferrous ion chelating activity compared to the Bambara groundnut (*Vigna subterranea*) protein hydrolysates, as reported by Arise et al. [38]. It also has a much higher ferrous ion chelating activity compared to phaseolin and bean protein hydrolysates, as reported by Carrasco-Castilla et al. [39] However, it has lower ferrous ion chelating activity compared to pea (*Pisum sativum* L.) protein hydrolysates, as reported by Pownall et al. [40] Thus, it can be said that mung bean albumin peptides are effective ferrous ion chelators. Of note, food-safe commercial enzymes such as alcalase and flavourzyme have been reported to produce hydrolysates with higher ferrous ion chelating activity compared to pepsin–pancreatin, as demonstrated by the sources above. These results are similar in this study, wherein thermolysin was found to produce peptides with higher iron ion chelating activity compared to pepsin–pancreatin, although the difference was also found to be not statistically significant.

Ascorbic acid was used as a standard in the ABTS assay as it is a water-soluble antioxidant present in foods. Previous publications also report the use of ascorbic acid as a standard for the ABTS assay [41].

The iron chelating and radical scavenging activities showed variation at different hydrolysis times for peptides derived via thermolysin and those derived via pepsin–pancreatin enzymatic hydrolysis (Figure S1). Overall, mung bean albumin peptides produced through hydrolysis by thermolysin showed higher radical scavenging and metal chelating activity, although the difference was not statistically significant at the end of hydrolysis. However, both types of peptides showed similar ORAC values.

According to the study on mung bean protein hydrolysates done by Sonklin et al. [42], radical scavenging activity was affected by both enzyme concentration and the hydrolysis time of a hydrolysate. Increasing enzyme concentration and hydrolysis time increased radical scavenging activity, but only until reaching a critical point, after which the activity became constant and decreased. As we used the same concentration of enzyme throughout the hydrolysis process, the variation in antioxidant capacity can be explained by the difference in hydrolysis time. Budseekoad et al. [26] reported that the iron ion binding capabilities of mung bean protein hydrolysates vary with hydrolysis time, and that different enzymes produce peptides with the highest calcium ion binding capabilities at different times. It can be theorized that different peptides with different antioxidant capacities were produced at different hydrolysis times, as was observed by this investigation.

Studies performed by Kong and Xiong [43] and Ajibola et al. [44] revealed that hydrophobicity played a part in the antioxidant capacity of peptides, with more hydrophobic peptides exhibiting a higher antioxidant capacity. Enzymatic hydrolysis using thermolysin produced mostly peptides with high hydrophobicity (>10 kcal/mol) compared to enzymatic hydrolysis using gastrointestinal enzymes, which explains the difference in the antioxidant capacity between the two types of hydrolysates analyzed. According to Zhu et al. [45], the size of peptides is a significant factor in their antioxidant capacity, whereby peptides with lower molecular mass exhibit higher antioxidant capacities. This assessment agreed with our results, as the thermolysin enzymatic hydrolysis produced peptides with lower molecular mass (Table 5).

Both high hydrophobicity and small molecular weight are factors that increase the absorption of peptides in the small intestine [46]. Peptides produced with thermolysin were of small molecular weight and of high hydrophobicity, indicating that they are expected to be absorbed during digestion. Antioxidant and hydrophobic casein peptides have already been shown to have good bioavailability using a Caco-2 cell model [47]. Future cellular studies are needed to measure the antioxidant capacity of mung bean protein-derived peptides before animal and human studies are performed.

## 5. Conclusions

Mung bean albumins at pH 7.0 have the same solubility as other mung bean proteins, making their extraction using pure water ineffective in isolating mung bean albumins from other proteins. Mung bean albumins at pH 8.3 had higher solubility than other mung bean proteins, making this pH better for mung bean albumin extraction. Mung bean albumin hydrolysates showed antioxidant potential in terms of ferrous ion chelating and ORAC values. They are particularly effective ferrous ion chelators. Hydrolysates produced via thermolysin enzymatic hydrolysis had higher antioxidant capacity overall due to their high hydrophobicity and low molecular mass. High hydrophobicity and low molecular mass are two factors that can increase the intestinal absorption of thermolysin-derived mung bean albumin peptides, but more in vivo studies are required to quantify their bioavailability in humans. The variation of antioxidant capacity over different time points during hydrolysis showed that different hydrolysis times produced different peptides of different antioxidant capacities. To our knowledge, this is the first study to investigate the antioxidant potential of mung bean albumin peptides through a variety of methods.

## Figures and Tables

**Table 1:** Possible Amino Acids at Positions in or Around Cleavage Site for Specific Proteases.

Protease	P4 Position	P3 Position	P2 Position	P1 Position	P1′ Position	P2′ Position	P3′ Position	P4′ Position
Alcalase	Gly, Pro, Ala, Val, Leu, Met, Phe, Tyr, Ser, Thr, Asn, Glu, His	Gly, Ala, Val, Leu, Phe, Ser, Thr, Asn, Gln, Asp, Glu, Lys, Arg, His	Gly, Pro, Ala, Val, Leu, Ile, Phe, Tyr, Ser, Thr, Asn, Gln, Glu, Arg, His	Ala, Val, Leu, Met, Phe, Tyr, Ser, Asn, Gln, Glu, Lys, Arg, His	Gly. Ala, Val, Leu, Met, Phe, Tyr, Ser, Thr, Gln, His	Gly, Pro, Ala, Val, Leu, Ile, Phe, Tyr, Ser, Thr, Gln, Glu, Arg, His	Gly, Pro, Ala, Val, Leu, Met, Phe, Tyr, Ser, Thr, Asn, Lys, Arg, His	Gly, Pro, Ala, Val, Leu, Tyr, Ser, Thr, Cys, Asn, Gln, Asp, Glu, Lys, His
Stem Bromelain	Pro, Ala, Leu, Ile, Phe, Tyr, Ser, Thr, Cys, Glu, Arg, His	Gly, Pro, Ala, Val, Leu, Phe, Ser, Thr, Lys, Arg, His	Gly, Pro, Val, Leu, Phe, Tyr, Ser, Thr, Asn, Arg, His	Gly, Ala, Val, Leu, Phe, Tyr, Ser, Thr, Asn, Gln, Arg	Gly, Val, Leu, Ile, Phe, Tyr, Ser, Thr, Asn, Gln, Asp, Glu, Lys, His	Pro, Ala, Val, Leu, Tyr, Ser, Thr, Asn, Gln, Asp, Glu, Lys, His	Gly, Pro, Val, Leu, Phe, Ser, Thr, Cys, Asn, Gln, Asp, Glu, Arg, His	Gly, Pro, Ala, Val, Leu, Phe, Tyr, Ser, Thr, Asp, Glu, Lys, Arg, His
Ficin	Pro, Leu, Ser, Glu, Lys, Arg, His	Gly, Ala, Val, Leu, Ile, Phe, Thr, Arg, His	Gly. Val, Leu, Phe, Thr, Lys	Gly, Leu, Phe, Tyr, Ser, Lys, Arg, His	Leu, Phe, Tyr, Ser, Thr, Lys, His	Val, Leu, Tyr, Thr, Asn, Lys, His	Pro, Ala, Val, Leu, Ser, Thr, Glu, Lys	Gly, Pro, Val, Asn, Asp, Glu, Lys
Papain	Gly, Pro, Ala, Val, Leu, Ile, Phe, Tyr, Ser, Thr, Cys, Asn, Asp, Glu, Arg, His	Gly, Pro, Ala, Val, Leu, Ile, Met, Phe, Tyr, Ser, Gln, Asp, Glu, Lys, Arg, His	All 20 Canonical AA	All 20 Canonical AA	Gly, Ala, Val, Leu, Ile, Met, Phe, Tyr, Ser, Thr, Asn, Gln, Asp, Glu, Lys, His	Gly, Pro, Ala, Val, Leu, Ile, Phe, Tyr, Ser, Thr, Cys, Asn, Gln, Glu, Arg, His	Gly, Pro, Ala, Val, Leu, Phe, Tyr, Ser, Cys, Glu, Lys, Arg, His	Gly, Pro, Ala, Val, Leu, Ile, Phe, Tyr, Ser, Thr, Asn, Asp, Glu, Lys, Arg, His
Thermolysin	All 20 Canonical AA	All 20 Canonical AA	All 20 Canonical AA	All 20 Canonical AA	Gly, Pro, Ala, Val, Leu, Ile, Met, Phe, Tyr, Trp, Ser, Thr, Asn, Gln, Asp, Glu, Lys, His	All 20 Canonical AA	All 20 Canonical AA	All 20 Canonical AA

AA = Amino Acids.

**Table 2:** Possible Small Antioxidant Peptides Produced from the Theoretical Hydrolysis of Mung Bean Albumin Sequences by Papain and Thermolysin.

Protease	Protein Sequence	Potentially Antioxidant Di-/Tri-Peptides Expected to be Produced ^1^	Number of Potentially Broken Antioxidant Fragments
Papain	Q9FRT8	GFC	0
	Q43680	EVY, VY, VYF, EAY, AY, AYV, VLK, KPS	6
Thermolysin	Q9FRT8	GFC, FC, FCI, LK, LKK, NFC	1
	Q43680	EVY, VY, VYF, EAY, AY, AYV, AYI, VYL, SIR, IR, IRN, AYL, VLK, LK, LKA, AFC, FC, EL, ELN, NKP, KP, KPS, NHL, HL, HLS	9

^1^ Potentially antioxidant fragments in the analyzed di-/tri-peptides are labeled in blue.

**Table 3:** Antioxidant Potential of Mung Bean Hydrolysates with Varying Hydrolysis Times.

Enzyme Scheme	Time (min)	ABTS Radical Scavenging Activity (Ascorbic Acid Equivalent, μM)	Iron Chelating Activity (EDTA Equivalent, μM)	ORAC (Trolox Equivalent, μM)
Thermolysin + MBA	0	685.0 ± 333.8	3034.8 ± 216.4	127 ± 19
	5	632.7± 288.6	5334.9 ± 45.3	125 ± 21
	10	683.2 ± 296.1	5337.8 ± 38.3	115 ± 17
	25	449.7 ± 301.4	5328.1 ± 17.4	98 ± 39
	45	511.0 ± 255.7	5329.2 ± 35.4	116 ± 17
	60	493.9 ± 297.7	5336.1 ± 26.2	127 ± 17
	240	646.2 ± 324.7	5161.7 ± 280.2	127 ± 17
	0	513.7 ± 230.4	3005.5 ± 248.0	122 ± 17
	15	533.5 ± 332.0	472.4 ± 227.4	126 ± 8
Pepsin + MBA	25	535.3 ± 328.0	529.4 ± 234.1	96 ± 20
	45	540.8 ± 307.3	500.2 ± 173.3	99 ± 35
	60	477.7 ± 316.6	490.7 ± 300.4	122 ± 12
	90	638.1 ± 340.2	594.3 ± 108.6	107 ± 42
	120	495.7 ± 342.3	434.3 ± 230.1	101 ± 46
	0	487.6 ± 338.4	448.5 ± 240.1	145 ± 38
	15	450.2 ± 283.0	4924.6 ± 119.4	105 ± 5
	25	474.1 ± 245.5	4901.0 ± 95.0	97 ± 31
Pepsin-Pancreatin + MBA	45	559.7 ± 197.0	4886.9 ± 182.8	121 ± 17
	60	456.9 ± 260.3	4884.4 ± 105.6	125 ± 16
	90	552.9 ± 256.4	4794.9 ± 16.5	124 ± 16
	120	581.3 ± 242.9	4843.5 ± 155.5	127 ± 15

MBA = Mung Bean Albumin; 1.0 mg/mL of EDTA is equivalent to 3421.8 μM; 1.5 mg/mL of EDTA is equivalent to 5132.8 μM; 0.15 mg/mL of ascorbic acid is equivalent to 851.7 μM; 1.0 mg/mL of ascorbic acid is equivalent to 5677.9 μM.

**Table 4:** Sequences and Functional Properties of Mung Bean Albumin Peptides Derived from Simulated Gastrointestinal Digestion.

Peptide Sequence	Molecular Mass (Da)	Hydrophobicity (Kcal/mol)	Isoelectric Point	Charge
MD	264	10.87	2.95	–1
QSA	304	9.63	5.49	0
EW	333	9.44	3.27	–1
LGW	374	5.71	5.69	0
KK	274	13.5	10.57	2
SVP	301	8.04	5.18	0
DVAF	450	9.87	3.05	–1

**Table 5:** Sequences and Properties of Mung Bean Albumin Peptides Derived from Thermolysin Enzymatic Hydrolysis.

Peptide Sequence	Molecular Mass (Da)	Hydrophobicity (Kcal/mol)	Isoelectric Point	Charge
KK	274	14.50	10.57	2
DM	264	10.87	3.02	–1
SY	268	7.65	5.38	0
W	204	5.81	5.64	0

## Supplementary Materials

The following are available online at https://www.mdpi.com/2304-8158/9/9/1241/s1, Table S1: Sequences, Functional Properties and Bioactivity of Mung Bean Albumin Hydrolysates Derived from Gastrointestinal Enzymatic Hydrolysis; Table S2: Sequences, Functional Properties and Bioactivity of Mung Bean Albumin Hydrolysates Derived from Thermolysin Enzymatic Hydrolysis; Figure S1: Antioxidant Potential of Mung Bean Albumin Hydrolysates at Various Hydrolysis Times from Two Different Enzyme Schemes. The star marks the time point at which pancreatin was added 2 h after pepsin. These results were obtained from hydrolysates derived from an average of 576 μg/mL of mung bean albumin.
