# Supplementary material for: Antioxidant Potential of Mung Bean (Vigna radiata) Albumin Peptides Produced by Enzymatic Hydrolysis Analyzed by Biochemical and In Silico Methods
Source: Foods. 2020 Sep 4;9(9):1241. doi: 10.3390/foods9091241 (PMC7554906; doi:10.3390/foods9091241)
Supplement: Supplementary file 1 [file foods-09-01241-s001.pdf]

Table S1. Sequences, Functional Properties and Bioactivity of Mung Bean Albumin Hydrolysates Derived from Gastrointestinal Enzymatic Hydrolysis.

Peptide Sequence	Molecular Mass (kDa)	Hydrophobicity (Kcal/mol)	Isoelectric Point	Charge
F	165.079	6.19	5.41	0
W	204.090	5.81	5.64	0
SM	236.083	7.69	5.33	0
ANG	260.112	10.40	5.61	0
EL	260.137	10.28	3.20	-1
MD	264.078	10.87	2.95	-1
KK	274.200	13.50	10.57	2
TTA	291.143	8.90	5.49	0
SVP	301.163	8.04	5.18	0
QSA	304.138	9.63	5.49	0
QSA	304.138	9.83	5.49	0
PEA	315.143	12.17	3.21	-1
SSK	320.169	11.62	9.88	1
EW	333.132	9.44	3.27	-1
APGP	340.174	9.83	5.24	0
SSNG	363.139	10.82	5.51	0
SCR	364.153	10.15	8.76	1
TPPG	370.185	9.58	5.49	0
LGW	374.195	5.71	5.69	0
GKTA	375.211	12.60	10.16	1
VSAT	376.195	8.65	5.36	0
CCR	380.130	9.67	8.30	1
GHTA	384.175	12.13	7.91	0
TSW	392.169	6.52	5.57	0
PPKG	397.232	12.13	10.59	1

AEW	404.169	9.94	3.27	-1
WGF	408.179	5.25	5.42	0
MGGY	426.157	8.82	5.39	0
PEW	430.185	9.58	3.27	-1
GDGW	433.159	11.75	3.18	-1
TGGSGG	434.176	13.21	5.49	0
HVW	440.217	7.68	7.69	0
LEW	446.216	8.19	3.27	-1
DVAF	450.211	9.87	3.05	-1
SKAF	451.242	9.95	9.88	1
EEW	462.175	13.07	3.09	-2
VCLE	462.214	9.80	3.09	-1
DGGGY	467.165	14.28	3.05	-1
SPEH	468.196	14.46	5.06	-1
TYW	468.200	5.35	5.56	0
GGSGKA	475.238	15.11	10.16	1
TDGW	477.185	10.85	3.18	-1
SMGW	479.183	6.75	5.60	0
PEGW	487.206	10.73	3.27	-1
VGGAW	488.238	8.15	5.69	0
YAPAA	491.237	8.83	5.50	0
AHGGPG	494.223	14.32	7.95	0
FFW	498.226	2.39	5.62	0
STKTA	506.269	12.16	9.88	1
TSDW	507.196	10.16	3.18	-1
SMSW	509.194	6.06	5.60	0
ESAGF	509.212	11.93	3.14	-1
SSPGY	509.212	9.40	5.38	0
YTGAT	511.227	9.34	5.29	0

THAGK	512.270	14.93	9.82	1
SPRGT	516.265	11.71	10.85	1
EAGGW	518.212	12.24	3.27	-1
SPPGPA	524.259	10.43	5.51	0
HEGW	527.212	12.92	5.06	-1
SSKGTG	535.259	14.17	9.88	1
MSDW	537.189	9.24	3.18	-1
STGGGY	540.217	11.35	5.38	0
HAEW	541.228	12.27	5.06	-1
GKGSGF	551.270	12.90	10.16	1
MSMW	553.202	4.93	5.61	0
YGMW	555.215	5.58	5.58	0
PYTW	565.253	5.49	5.72	0
ECVGY	569.215	11.49	3.14	-1
SPKAAP	569.316	12.44	9.88	1
TPPALS	584.316	8.14	5.36	0
STGGGSM	595.226	11.85	5.33	0
PQVAW	599.306	6.76	5.75	0
DSAEW	606.228	14.04	3.05	-2
YGMGW	612.236	6.73	5.58	0
STGVAW	619.296	7.71	5.60	0
AGKGSFG	622.307	13.40	10.21	1
PGLSAW	629.316	6.81	5.75	0
TPAAATT	631.317	10.29	5.28	0
NPSLGTG	644.312	10.65	5.38	0
VPPGKGGP	707.396	14.11	10.14	1
STGYTW	713.301	7.21	5.58	0
SPKPGAGL	725.406	12.99	9.88	1
TEGGFCD	727.247	15.99	2.82	-2

TPKGGSGK	730.396	17.80	10.57	2
TSKSMW	738.336	9.11	9.82	1
SRGGGAGW	746.345	13.18	10.85	1
SSKGPAASG	760.370	15.52	9.88	1
TPPPGKTA	767.417	13.27	9.82	1
SPPAGGPTL	795.411	10.58	5.50	0
CSVFSAW	798.336	5.04	5.29	0
TSYGTTW	814.349	7.46	5.56	0
KTGAGAGAAL	815.449	15.15	9.80	1
TPKAGGGPGV	839.449	15.87	9.82	1
TPGLGGGKTA	857.459	15.19	9.82	1
TPPVGGGKTA	883.475	14.97	9.82	1
SPKTPGGATA	885.454	15.24	9.88	1
KTGPSGASKG	888.465	18.76	10.57	2
QPGGGGGGGGAR	926.431	20.32	10.80	1
GHPAGTAATR	937.472	16.48	11.13	1
QPSVGPPAAF	969.491	9.53	5.38	0
SPKPGPPVSL	977.553	11.62	9.88	1
SPKPGPPVGTA	1006.543	14.31	9.88	1
TPKGPASPGHV	1046.549	16.50	9.82	1
LSPAKPPGVSKG	1136.653	15.93	10.64	2
YPPEVGAGSGKSAW	1404.665	16.72	6.58	0
TPRGGGDSPPPLCTR	1509.734	18.86	8.70	1

$^{+}$ Indicates positive antioxidant capacity according to the BIOPEP database.

Table S2. Sequences, Functional Properties and Bioactivity of Mung Bean Albumin Hydrolysates Derived from Thermolysin Enzymatic Hydrolysis.

Peptide Sequence	Molecular Mass (Da)	Hydrophobicity (Kcal/mol)	Isoelectric Point	Charge
W	204.090	5.81	5.64	0
ANG	260.112	10.40	5.61	0
DM	264.078	10.87	3.02	-1
SY	268.106	7.65	5.38	0
KK	274.200	14.50	10.57	2
TTA	291.143	8.90	5.49	0
GDM	321.099	12.02	3.02	-1
ASR	332.180	10.67	11.18	1
ATR	346.196	10.46	11.18	1
SSR	348.175	10.63	10.85	1
GGGY	352.138	10.64	5.45	0
TGW	362.159	7.21	5.57	0
CEN	364.105	12.36	3.11	-1
PPGV	368.205	8.87	5.69	0
GKTA	375.211	12.60	10.16	1
AFR	392.217	8.50	11.18	1
EDM	393.120	14.50	2.89	-2
TTEA	420.185	12.53	3.21	-1
GNMD	435.142	12.87	2.95	-1
TTKS	435.232	11.66	9.82	1
ATMD	436.162	11.62	2.95	-1
STDM	452.157	11.58	3.02	-1
LHLA	452.274	8.23	7.89	0
SNGW	462.186	8.27	5.60	0
DTDM	480.152	14.76	2.85	-2
TTKF	495.269	9.49	9.82	1
MTDM	496.166	10.45	3.02	-1

NGGGTM	535.205	11.78	5.22	0
HASGPA	538.249	12.98	7.69	0
TTKSGG	549.275	13.96	9.82	1
GKSGGF	551.270	12.90	10.16	1
NGGGSY	553.213	11.95	5.27	0
NNGPR	556.271	12.70	10.60	1
HTGGAGA	569.255	14.93	7.69	0
SPKPAA	569.316	12.44	9.88	1
SPPLGT	570.300	8.79	5.30	0
EAAAPW	643.296	11.08	3.27	-1
HASGSSR	700.324	15.07	10.91	1
SPHGASR	710.345	14.75	10.85	1
TPPAAGPSS	783.375	11.64	5.36	0
ASAGPGSSR	788.377	14.53	11.18	1
SVAVGGKTA	788.438	13.79	9.88	1
TPPPGAGSR	838.429	13.64	10.79	1
KESGGGVGR	845.434	20.74	9.80	1
THGGPAGTGP	850.392	16.11	7.57	0
YMMDM	869.221	8.82	3.02	-1
HTGSPAGALA	880.439	13.63	7.69	0
TTGTGGPAGGY	937.413	14.33	5.36	0
EKGGSGVGGGW	989.455	19.14	6.71	0
SSKGGGPAAGAEW	1173.540	19.40	6.60	0

[+] Indicates positive antioxidant capacity according to the BIOPEP databas.

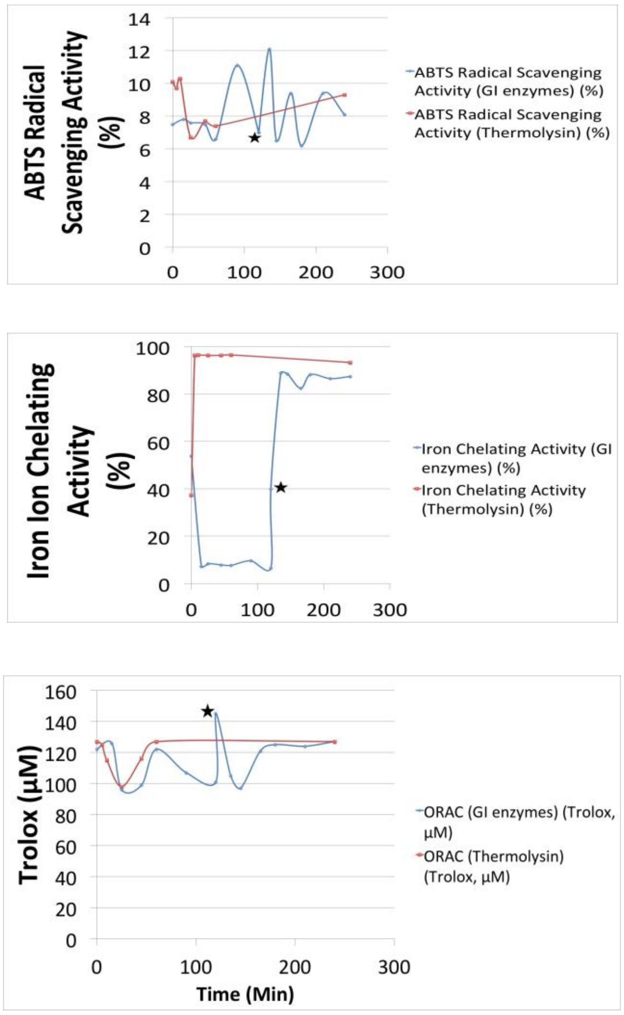

Figure S1. Antioxidant potential of mung bean albumin hydrolysates at various hydrolysis times from two different enzyme schemes. The star marks the time point at which pancreatin was added 2 h after pepsin. These results were obtained from hydrolysates derived from an average of 576 µg/mL of mung bean albumin.
